# A three-tiered mentorship approach for supporting high school students interested in science, technology, engineering, and mathematics (STEM) careers

**DOI:** 10.1017/cts.2025.19

**Published:** 2025-02-10

**Authors:** Brittney D. Browning, Janiece S. Glover, Lindsay R. Meredith, Anna E. Kirkland, Kathryn S. Gex, Rachel L. Tomko, Felicity Duong, Lindsay M. Squeglia

**Affiliations:** 1 Department of Psychiatry and Behavioral Sciences, Medical University of South Carolina, Charleston, SC, USA; 2 Department of Pathology, Microbiology and Immunology, Vanderbilt University Medical Center, Nashville, TN, USA; 3 Hollings Cancer Center, Medical University of South Carolina, Charleston, SC, USA; 4 The Matilda Centre for Research in Mental Health and Substance Use Research, University of Sydney, Sydney, Australia

**Keywords:** Mentoring, stem, high school, near-peer, youth, adolescent, diversity, underrepresented minorities

## Abstract

Racially and ethnically minoritized individuals, first-generation college students, and women are significantly underrepresented in science, technology, engineering, and mathematics (STEM) careers. This lack of equal representation limits creativity and progress in these fields and perpetuates systemic barriers that discourage students from pursuing STEM pathways. This special communication introduces the three-tiered mentorship model employed in the Teen Science Ambassador Program (TSAP), which incorporates senior mentors, near-peer mentors, and high school ambassadors (i.e., mentees) to promote education, hands-on research, and career development in STEM for underrepresented students. We discuss the benefits and challenges of the three-tiered model and offer recommendations for optimizing its effectiveness to enhance mentorship experiences for all participants. Findings from the TSAP program suggest that the three-tiered approach benefited all participants: high school ambassadors gained STEM skills and confidence, near-peer mentors developed leadership and communication abilities, and senior mentors improved mentorship skills. However, the effectiveness of near-peer mentorship is highly dependent on clearly defined roles and structured involvement. Thus, feedback collected from each mentorship tier was used to inform subsequent iterations of the program. The layered mentorship structure fostered a sense of community and belonging, which is crucial for retaining individuals from underrepresented groups in STEM.

## Introduction

Diversity is a key driver of innovation, yet there is a significant underrepresentation of racially and ethnically minoritized individuals, first-generation college students, and women in science, technology, engineering, and mathematics (STEM) careers [1]. This lack of equal representation not only stifles creativity and progress within these fields but also perpetuates systemic inequalities that discourage students from pursuing STEM. There has been significant growth in the number of STEM mentorship programs in the United States; however, individuals who are traditionally underrepresented in STEM fields may be less likely to know about or have access to these opportunities [2,3]. To address this, many STEM mentoring programs have created initiatives to recruit youth from underrepresented groups to participate in their programs. Despite this, many underrepresented groups continue to face challenges in accessing high-quality mentorship, networking opportunities, and career advancement in STEM. To address these challenges, it is essential to implement novel approaches that foster inclusivity and engagement among underrepresented groups, as these factors have been shown to be powerful predictors of retention and success in STEM careers [3].

Mentoring is widely recognized for its positive impact on individuals pursuing careers in STEM, particularly for those from underrepresented groups. Effective mentoring relationships provide essential guidance, support, and resources, helping mentees navigate academic challenges and fostering their confidence and sense of belonging within these fields. For underrepresented populations, mentoring has been shown to increase opportunities, promote equity, and reduce attrition by addressing specific obstacles such as stereotype threat, limited role models, and lack of support [4]. Research has demonstrated that mentoring can significantly enhance scientific identity, increase academic achievement, improve retention, and lead to greater persistence in STEM studies among individuals underrepresented in science [2,5]. Nonetheless, there is large variability in the impact of youth mentoring programs aimed at increasing diversity in STEM, and effective strategies are needed to maximize their impact.

In a traditional STEM mentorship model, a senior mentor is paired with a student. Near-peer mentoring is another type of mentoring model, where mentors are closer in age but slightly more advanced in their academic journeys than their mentees [6,7]. Adding near-peer mentors to the traditional mentorship model may enhance the effectiveness of mentoring programs, particularly in regard to fostering a sense of scientific community and belonging. Near-peer mentors are often more relatable than established senior mentors due to their recent experiences, making them more approachable and better able to provide practical, relevant advice. Their proximity in age and career stage helps create a more comfortable environment for mentees to ask questions and seek guidance without feeling intimidated. Near-peer mentorship is also highly beneficial for the near-peer mentors, as it instills confidence and reinforces previously learned STEM concepts. This mutual support cultivates stronger mentor–mentee relationships and builds scientific identity, ultimately supporting long-term retention and success in STEM fields.

**Figure 1.**
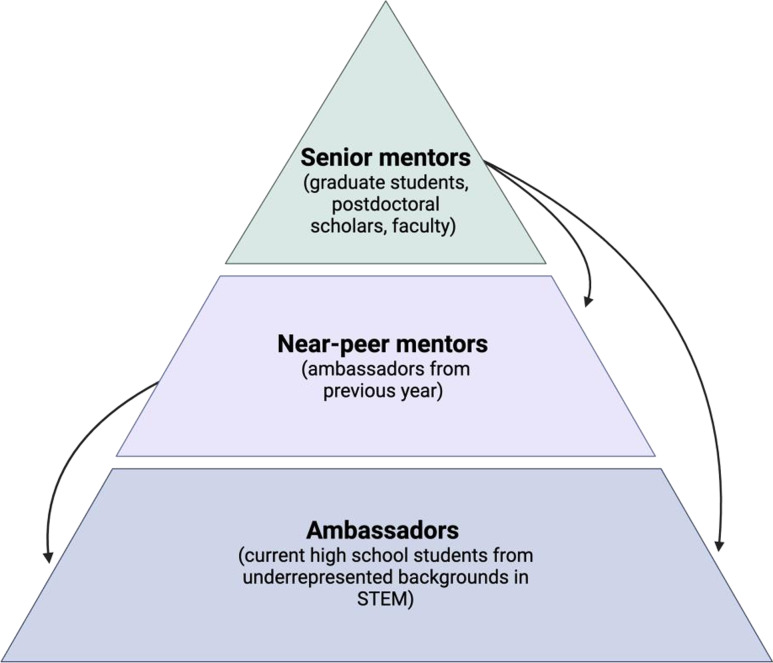
Mentoring structure. Senior mentors, which consist of graduate students, postdocs, and faculty, provide mentorship to both near-peer mentors (ambassadors from the previous year) and ambassadors (current mentees that are high school students from underrepresented backgrounds in STEM). Near-peer mentors both receive mentorship from senior mentors and provide mentorship to current ambassadors.

In response to the need for more effective mentorship programs for underrepresented students, we modified our Teen Science Ambassador Program (TSAP), which equips high school students with education, hands-on research experience, and mentoring in STEM fields, to incorporate a three-tiered mentorship approach [8]. In this approach, each mentee (ambassador) is connected with both a near-peer mentor (a former ambassador) and a senior mentor (graduate student, postdoctoral scholar, or faculty member). Importantly, this model fosters a layered system of reciprocal mentorship: Ambassadors receive guidance from both near-peer and senior mentors, while near-peer mentors both provide mentorship to ambassadors and receive mentorship from senior mentors (Fig. 1). This paper explores how the three-tiered mentorship model implemented in TSAP can serve as a framework for enhancing mentorship in STEM education, fostering not only scientific interest but also increased confidence and a sense of belonging among underrepresented youth.

## TSAP program

**Figure 2.**
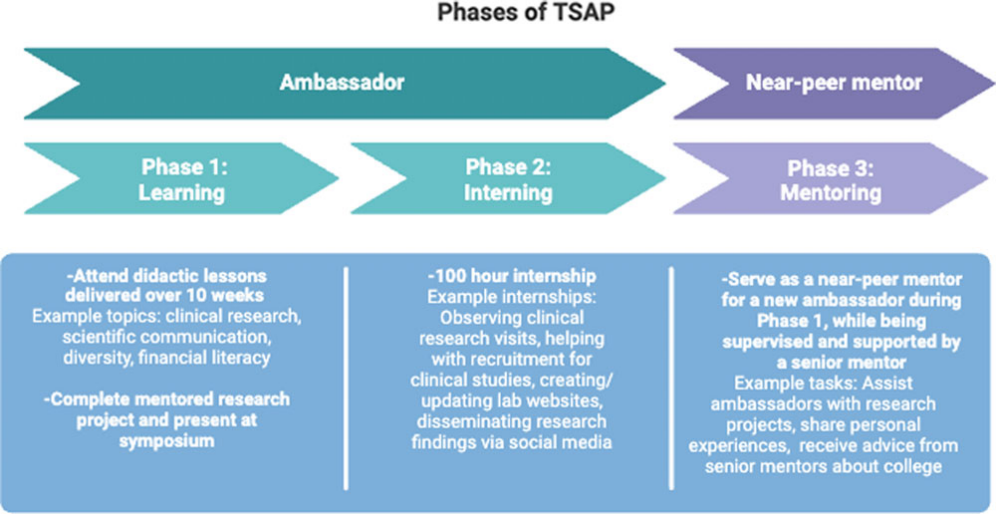
Three-phase structure of the Teen Science Ambassador Program (TSAP).

The TSAP is a two-year program to engage high school students in STEM careers, specifically in the fields of mental health and substance use (Margherio et al., 2024). The program took place after school, and the ambassadors were compensated $20 per hour for their participation during each phase of the program. The first phase of the program includes 10 weeks of interactive lessons that introduce research and skills that are essential for pursuing careers in STEM. Ambassadors are also guided through an inquiry-based research project with their mentors, helping them develop personalized research questions, conduct literature reviews, and present their findings in a mini-symposium [8]. The second phase of the program consists of a paid 100-hour internship on National Institutes of Health funded research projects. Finally, during the third phase of the program, ambassadors transition into near-peer mentors. Figure 2 displays the three-phase structure of TSAP. We invite interested readers to explore our TSAP resource bank (https://sites.google.com/view/tsap-resources/home) and previous publication [8], where they can find detailed information about program coordination and content, including specific guidelines to facilitate program replication.

## Mentoring structure

Ambassadors (i.e., mentees) consist of local high school students that meet NIH’s definition for groups traditionally underrepresented in biomedical science research, including individuals from racial and ethnic groups underrepresented in science, individuals with disabilities, those from disadvantaged backgrounds, and women. We specifically targeted sophomores and juniors in high school for this program due to their critical developmental stage, where exposure to STEM can significantly influence their educational and career trajectories. The goal is to inspire a diverse new generation of scientists by providing early exposure to research opportunities that they might not otherwise encounter. Importantly, academic performance (e.g., GPA) is not part of the application, and individuals with no prior STEM experience are encouraged to apply, emphasizing the program’s focus on student potential and interest over prior achievements.

Near-peer mentors are previous ambassadors who have successfully completed the ambassador phase of the program. Near-peers experience both sides of the mentor–mentee relationship; they are mentored by senior mentors while concurrently mentoring their own mentees (ambassadors). This process ensures that near-peer mentors represent individuals from groups underrepresented in the biomedical sciences and have a deep familiarity with the program’s goals and structure. Near-peer mentors bridge the gap between senior mentors and mentees, offering relatable guidance and fostering a sense of belonging. They share their own experiences and challenges, providing mentees with practical insights and strategies for navigating their academic and professional journeys. Near-peer mentors undergo a 2-hour hybrid training, combining self-paced online modules and face-to-face instruction. The training is based on the Ready to Go: Mentor Training Toolkit designed to equip mentors with the necessary skills to foster strong, productive relationships with their mentees [9]. In addition, they are provided with the Peer Mentoring Handbook, which outlines expectations for mentoring younger students and offers best practices for building high-quality peer mentoring relationships, as provided by the Mentoring Partnership of Southwestern [10].

Senior mentors consist of graduate students, postdoctoral scholars, and faculty members who are actively engaged in mental health and/or substance use research. They play a pivotal role in the professional development of both mentees and near-peer mentors by offering expert guidance, sharing knowledge, and modeling effective research and mentorship practices. Their responsibilities include providing high-level research instruction, teaching essential skills, and offering career advice. To ensure consistency and quality, senior mentors participate in a 2-hour training session where they review program expectations and best practices for mentorship.

## Outcomes and impact

Both qualitative and quantitative evaluations were employed to gather insights from TSAP participants, including ambassadors (after phase 1), near-peer mentors, and senior mentors. Qualitative data gathered from focus groups led by external evaluators were transcribed and examined through memos to identify emerging themes, serving both as formative and summative feedback [11]. The Teen Ambassadors Outcomes Survey was created by the project study team to specifically target two critical areas given their particular benefits in supporting individuals underrepresented in biomedical science: STEM career interest, assessed through items from the STEM Career Interest Survey (CIS) [12] and STEM belonging, growth mindset, and identity, measured using the STEM-Belonging, Growth Mindset, and Identity (BGI) scale [13].

The first cohort of the program followed a simpler mentoring structure, consisting solely of ambassadors paired with senior mentors. In contrast, cohorts 2 and 3 introduced the tiered model, incorporating near-peer mentors alongside senior mentors and ambassadors. This evolution in the mentoring approach allows for a comparison across cohorts, enabling an assessment of how effective the tiered model is in fostering ambassador outcomes, including interest, confidence, and sense of belonging.

## Ambassador outcomes

Demographics for ambassadors from cohorts 1–3 are provided in Table 1. Across all three cohorts, ambassadors consisted mostly of students who identified as Black/African American females. All ambassadors attended at least 80% of the classes.

**Table 1.** Demographics of ambassadors from cohorts 1–3

Demographics	Cohort 1 (*n* = 6)	Cohort 2 (*n* = 11)	Cohort 3 (*n* = 11)
**Gender**			
Male	1 (17%)	1 (9%)	1 (9%)
Female	5 (83%)	10 (91%)	10 (91%)
**Race**			
Black/African American	4 (67%)	8 (73%)	6 (55%)
White/Caucasian	2 (33%)	2 (18%)	2 (18%)
Asian	–	1 (9%)	2 (18%)
Other	–	–	1 (9%)
**Ethnicity**			
Hispanic/Latino	3 (50%)	1 (9%)	2 (18%)
**Grade**			
10th	3 (50%)	7 (64%)	10 (91%)
11th	3 (50%)	4 (36%)	1 (9%)

Qualitative feedback from ambassadors highlighted the strong connections formed with their mentors, which led to high engagement, increased interest in science, and a sense of anticipation for future STEM opportunities. Ambassadors also reported feeling more confident in their ability to pursue STEM-related academic and career paths, often attributing their motivation to the support and encouragement provided by their mentors.

The quantitative data from the CIS further supports this increased interest and confidence in STEM, with all cohorts showing higher post-scores (Table 2). Cohort 1, which included only senior mentors, had the most variability in CIS total score, as indicated by its higher SD (4.12 at pre-test and 3.16 at post-test) and a mean change score of 3.00 (SD = 2.45). Cohort 2 also experienced increases in their total scores, but these gains were not as pronounced, resulting in a mean change score of 1.55 (SD = 3.08) and pre-score of 15.64 (SD = 2.46). This cohort faced challenges as it was the first time implementing the mentorship model with both senior and near-peer mentors. Finally, cohort 3 demonstrated the most effective outcomes in the CIS, with every pre- and post-score, including the total CIS score, being significantly different, indicating significant growth in ambassadors’ career interests. Notably, the total CIS pre-scores for cohort 3 were lower than those of the other cohorts, with a mean pre-score of 13.27 (SD = 2.10). This may reflect a trend of declining interest in science among high school students, but it may also be influenced by recruitment factors or the small sample size, which should be considered when interpreting the results.

**Table 2.** Pre-post outcomes and change scores from teen ambassadors outcomes survey (CIS and BGI) across cohorts 1–3

Outcomes	Cohort 1 (no near-peer mentors),			Cohort 2,			Cohort 3,		
	*n* = 6			*n* = 11			*n* = 11		
	Pre mean (SD)	Post mean (SD)	Mean Change Score (SD)	Pre mean (SD)	Post mean (SD)	Mean Change Score (SD)	Pre mean (SD)	Post mean (SD)	Mean Change Score (SD)
CIS: I plan to use science in my future career. (1–5)	4.17 (0.75)	4.67 (0.52)	0.5 (0.84)	4.27 (0.79)	4.36 (0.92)	0.09 (0.83)	3.64 (0.67)	4.64 (0.67)*	1 (0.63)
CIS: I am interested in careers that use science. (1–5)	4.17 (1.17)	4.50 (0.84)	0.33 (0.82)	4.00 (1.00)	4.09 (1.04)	0.09 (0.30)	3.82 (0.60)	4.82 (0.40)*	1 (0.63)
CIS: I have a role model in a science career. (1–5)	3.33 (1.63)	4.00 (1.55)	0.67 (0.82)	3.09 (1.14)	3.45 (1.21)	0.36 (1.50)	2.18 (1.08)	3.91 (1.30)*	1.73 (1.49)
CIS: I would feel comfortable talking to people who work in science careers. (1–5)	4.17 (1.17)	4.83 (0.41)	1.5 (1.38)	4.27 (0.65)	4.09 (0.83)	1 (1.34)	3.64 (0.81)	4.36 (0.67)*	2.18 (1.17)
CIS total score	15.83 (4.12)	18.00 (3.16)	2.17 (2.14)	15.64 (2.46)	16.00 (2.93)	0.36 (2.01)	13.27 (2.10)	17.73 (2.00)*	4.45 (2.21)
BGI: With the right amount of effort/dedication, anyone can become a top STEM scholar. (1–6)	5.00 (2.00)	5.00 (2.00)	0 (0.63)	5.36 (0.67)	5.55 (0.82)	0.18 (0.60)	4.73 (0.79)	5.55 (0.69)*	0.82 (0.87)
BGI: I see myself as a STEM person. (1–6)	4.33 (2.07)	4.83 (2.04)	0.5 (0.84)	4.82 (0.98)	5.18 (0.75)	0.36 (0.67)	4.00 (1.00)	5.27 (0.79)*	1.27 (1.27)
BGI: People like me belong in STEM careers. (1–6)	4.50 (1.87)	5.00 (2.00)	0.5 (0.84)	4.82 (0.75)	5.00 (0.89)	0.18 (0.60)	4.82 (1.08)	5.73 (0.65)*	0.91 (1.04)
BGI: Other people I know see me as a STEM person. (1–6)	3.83 (1.72)	4.83 (2.04)*	1 (0.89)	4.33 (1.21)	4.73 (1.19)	0.36 (0.67)	3.82 (0.98)	4.91 (0.83)*	1.09 (0.70)
BGI total score	17.67 (7.31)	19.67 (8.04)	2.00 (2.68)	19.36 (2.94)	20.45 (3.27)	1.09 (1.58)*	17.36 (2.20)	21.45 (2.86)*	4.09 (2.51)

CIS = Career Interest Survey; BGI = Belonging, Growth Mindset, and Identity Scale.

Collected from ambassadors after phase 1 of the program. Significant differences between pre- and post-means were determined using a paired *t*-test, with * indicating significance at *p* < 0.05.

Qualitative data provided additional insights. Ambassadors emphasized the positive impact of seeing mentors who shared similar backgrounds and experiences, which increased their sense of belonging and hope for achieving their goals. One ambassador from cohort 2 said,

It was really nice to see people that you can look up to that are like you, and you know, you don’t see those types of people in your field. So, for someone to be there, it kind of gives you hope that I can do this. I can make it; I can be just like them!

Another ambassador from cohort 1 had a similar sentiment and stated,

I feel growing up there weren’t as many like figures we could relate to … now you’re able to talk to someone who like is able to understand your struggles and like how they’ve been able to deal with it and how they are able to… manage their time and like be successful in their fields despite you know, their backgrounds.

This increased sense of belonging was also observed using quantitative data. In all three cohorts, higher post-scores were observed on the BGI scale questions (Table 2). Cohort 1 displayed the highest variability in total BGI scores, with a standard deviation of 7.31 at pre-test and 8.04 at post-test. This variability suggests that the absence of near-peer mentors may have made it more challenging to foster a consistent sense of belonging among ambassadors. The post-mean score for the BGI item, “Other people I know see me as a STEM person,” was the only item significantly higher than the pre-mean score (*p* = 0.041). Cohort 2 also experienced positive shifts in total BGI scores, with a mean change score of 1.09 (SD = 1.58) and a pre-score of 19.36 (SD = 2.94). The pre- and post-total BGI scores were significantly different for cohort 2 (*p* = 0.045). Cohort 3 showed the most significant gains in BGI, displaying significantly different pre- and post-scores on every BGI item, including the total score, suggesting an enhanced sense of belonging and identity within the STEM field (*p* < 0.05). Ambassadors in cohort 3 began with a lower total BGI score of 17.36 (SD = 2.20), potentially indicating a need for stronger support in establishing a sense of connection to STEM. However, the structured support and role clarity in cohort 3 allowed for a meaningful increase in ambassadors’ perceived belonging and growth mindset. Overall, the significant differences in pre- and post-total BGI scores for cohorts 2 and 3 support the effectiveness of the tiered mentorship model in reinforcing ambassadors’ connections to the STEM community.

In terms of the three-tiered mentorship approach, ambassadors generally described stronger relationships with their senior mentors in comparison to their near-peer mentors, which may be due to ambassadors perceiving quietness and lack of proactiveness on the part of the near-peers. Some ambassadors felt their near-peer mentors were not as helpful in research but knew more about the structure of the final research presentation.

## Near-peer mentor qualitative feedback

In cohorts 2 and 3, all near-peer mentors appreciated the leadership opportunities provided by the program. Although we did not directly measure skill acquisition, near-peers noted that these experiences boosted their confidence and developed their leadership skills. Near-peers enjoyed their role, connected well with ambassadors on a personal level, and reported, “overall it was a really good experience.” The evaluation team also noted evidence of growth in near-peers’ confidence and comfort in terms of the speed, tone, and enthusiasm they communicated compared to the first time near-peers participated in a focus group. Further, near-peers expressed thoughtful post-secondary plans and valued networking opportunities provided through TSAP. Near-peer mentors also commented on the role helping with their leadership skills. For instance, one near-peer from cohort 3 stated, “I’ve become more confident and having those leadership skills like kind of boosted that.”

Despite positive feedback, near-peers expressed uncertainty about their roles at times, particularly when ambassadors were focused on independent research. Others agreed with a near-peer who summarized, “I would say one of the challenges…is that there were times when it just felt not as necessary for me to be right there at that moment just because they’re in their independent work.” Adjustments were made to more clearly define near-peer roles based on feedback from cohort 2. It is important to note that near-peers in cohort 2 did not have near-peers themselves to serve as an exemplar while they were ambassadors. While there were improvements on role clarification in cohort 3, near-peers in this cohort also expressed the desire for more defined roles when working with ambassadors one-on-one. They explained, “what will help with [being included] is having more really specific roles for helping out with meetings, but we didn’t really have specific roles for helping out with their projects.” Some near-peers were able to bond well with their ambassadors, while others reported feeling more “detached.” One near-peer from cohort 3 explained how at first “everybody was really timid. But … by the end, people felt more comfortable to ask questions and [they] asked what was really on their mind.”

## Senior mentor qualitative feedback

Senior mentors provided valuable guidance and support to both ambassadors and near-peer mentors. Many expressed how rewarding it is to witness the growth in their mentees’ confidence over time. One senior mentor from cohort 1 said,

[My favorite was] those personal moments where you really connect and you can see them grow and [they’re] becoming more confident, especially after they practiced their presentation. They were like, oh, I know this. Yeah, you know this! And just being able to have that mentor relationship I think, because it’s like something that I always had trouble finding, especially in high school, because of my background and being able to provide that for someone else.

The progression from ambassador to near-peer mentor also allowed senior mentors to witness leadership growth. One senior mentor from cohort 2 reflected on the development of a near-peer mentor, stating,

She also was always confident, but I remember when she first met the mentees. She went up to them and was like, this program was so great, and I learned all these things and [was] just talking like she was one of the mentors! Just super confident and like she knew everything about the program. It was just awesome to see her growth because I could not have seen her doing that last year.

In addition to observing growth in the mentees, the transition from ambassador to near-peer mentor allows senior mentors to form stronger relationships with them. Some senior mentors even expressed a desire to continue supporting mentees beyond the TSAP program, suggesting a deeper commitment to the mentees’ development and well-being. One stated they wanted to be “a mentor for them like past TSAP… and just continue to have those connections after they leave. I would like that at least.”

Senior mentors spoke about the benefits of the “team-based mentoring approach,” where senior mentors were paired with each other to oversee multiple near-peers and ambassadors. For new senior mentors, they were paired with previous TSAP mentors, which was particularly valuable for new senior mentors in the program. The model was perceived as especially beneficial when someone was absent so that no one was “left in the dark.” They also mentioned that the team model allowed for greater support and natural connections among team members. Despite this, senior mentors also noted a challenge with balancing time and attention between the near-peer mentor and mentee. One reflected there were “definitely times where I didn’t quite involve the peer mentor enough… you have to think about how to balance that time with their roles so they feel like they can be part of the process too.” Another agreed they “felt like we were not able to utilize [near-peer mentors] well because I didn’t know where they were going to fit in actually. But after we had the second check-in, it got a little bit easier.” These check-ins were structured meetings designed to provide mentors with opportunities to share their experiences and challenges in real time. This allowed for immediate feedback and adjustments, helping mentors better understand how to effectively integrate the near-peer mentors into the program.

## Discussion/recommendations

These finding suggests that the three-tiered mentorship approach is advantageous for all participants, including ambassadors, near-peer mentors, and senior mentors. For ambassadors, the guidance from mentors who have recently navigated similar academic and career paths provides relatable support and a clearer understanding of what is needed to succeed in STEM fields. Near-peer mentors benefit by reinforcing their own knowledge through teaching, enhancing leadership skills, and gaining a sense of purpose by giving back to the STEM community. Senior mentors, on the other hand, experience growth in their mentorship skills while fostering a collaborative and supportive learning environment that promotes continuous professional development for all involved.

The effectiveness of the three-tiered mentorship approach can be maximized with clarity of roles. While ambassadors in cohort 2 benefited from near-peer mentoring, the impact was much stronger in cohort 3, likely due to the introduction of clearer roles based on feedback from focus groups. It is important to note that cohort 2 started with the highest total pre-scores, which may have limited their potential for growth compared to cohort 3, who had more room for improvement. In fact, the average total scores for cohort 3 eventually surpassed cohort 2, suggesting that the mentorship model may be particularly effective for those with more room for growth. Cohort 2, being the first to implement near-peer mentoring, experienced more role ambiguity and lacked the guidance of previous near-peers to learn from. Despite the improvements, cohort 3 still expressed a desire for more clearly defined roles when working one-on-one with ambassadors.

Providing near-peer mentors with a set of probing questions or prompts they can use to engage ambassadors during research activities such as asking about challenges, next steps, or offering constructive feedback might be one strategy. Senior mentors also expressed not knowing how to include the near-peers at times, so they could also be provided with a set of probing questions to get near-peers involved if the near-peer is not taking the initiative. Another approach would be to foster stronger personal connections between all participants. Starting off with an ice breaker at the beginning of sessions is a good way to get mentees to open up. It is also key that all program participants (ambassadors, near-peer mentors, and senior mentors) have a clear understanding of each other’s roles. It is recommended to schedule regular debrief sessions between near-peers and senior mentors to discuss their roles, challenges, and successes. This provides a space for near-peers to reflect on their experience, ask for advice, and adjust their approaches as necessary. These regular check-ins and continued leadership opportunities will also ensure that near-peers feel integrated and valued throughout the program, helping them stay connected both to the ambassadors and to the larger mentoring team.

While the evolution in the mentoring approach across the 3 cohorts provides an advantageous opportunity to compare the simpler model of cohort 1 with the tiered model implemented in cohorts 2 and 3, it also presents a limitation. The inherent differences between the mentoring structures may introduce variability that is difficult to control, as the cohorts were not subjected to the same mentoring conditions. Additionally, it would be valuable to examine how ambassador characteristics, such as racial/ethnic identity, sex distribution, and grade level, may influence outcomes; however, our current study lacks the statistical power to reliably assess these factors. Future research with larger sample sizes is needed to explore potential differences in outcomes based on these factors.

## Conclusion

Based on the qualitative and quantitative feedback received in the TSAP program, the three-tiered mentorship approach appears to be a promising strategy for addressing the urgent need to promote diversity in STEM careers. By involving individuals at different stages of their academic and professional journeys, this model not only supports mentees but also empowers mentors to develop leadership skills and gain new perspectives. It creates a cycle of growth and learning that encourages long-term engagement and investment in STEM fields. Moreover, the layered mentorship structure fosters a sense of community and belonging, which is crucial for retaining individuals from underrepresented groups in STEM.
